# Curcumin: Novel Treatment in Neonatal Hypoxic-Ischemic Brain Injury

**DOI:** 10.3389/fphys.2019.01351

**Published:** 2019-11-13

**Authors:** Eridan Rocha-Ferreira, Claudia Sisa, Sarah Bright, Tessa Fautz, Michael Harris, Ingrid Contreras Riquelme, Chinedu Agwu, Tugce Kurulday, Beenaben Mistry, Daniel Hill, Sigrun Lange, Mariya Hristova

**Affiliations:** ^1^Department of Maternal and Fetal Medicine, Perinatal Brain Repair Group, UCL Institute for Women’s Health, London, United Kingdom; ^2^Department of Neuroscience and Physiology, Sahlgrenska Academy, University of Gothenburg, Gothenburg, Sweden; ^3^Department of Molecular Biology and Genetics, Izmir Institute of Technology, İzmir, Turkey; ^4^Department of Visual Neuroscience, Glaucoma and Retinal Neurodegeneration Group, UCL Institute of Ophthalmology, London, United Kingdom; ^5^School of Life Sciences, Tissue Architecture and Regeneration Research Group, University of Westminster, London, United Kingdom

**Keywords:** curcumin, hypoxia, ischemia, neuroprotection, neonate, oxidative stress

## Abstract

Hypoxic-ischemic encephalopathy (HIE) is a major cause of mortality and morbidity in neonates, with an estimated global incidence of 3/1,000 live births. HIE brain damage is associated with an inflammatory response and oxidative stress, resulting in the activation of cell death pathways. At present, therapeutic hypothermia is the only clinically approved treatment available for HIE. This approach, however, is only partially effective. Therefore, there is an unmet clinical need for the development of novel therapeutic interventions for the treatment of HIE. Curcumin is an antioxidant reactive oxygen species scavenger, with reported anti-tumor and anti-inflammatory activity. Curcumin has been shown to attenuate mitochondrial dysfunction, stabilize the cell membrane, stimulate proliferation, and reduce injury severity in adult models of spinal cord injury, cancer, and cardiovascular disease. The role of curcumin in neonatal HIE has not been widely studied due to its low bioavailability and limited aqueous solubility. The aim of this study was to investigate the effect of curcumin treatment in neonatal HIE, including time of administration and dose-dependent effects. Our results indicate that curcumin administration prior to HIE in neonatal mice elevated cell and tissue loss, as well as glial activation compared to HI alone. However, immediate post-treatment with curcumin was significantly neuroprotective, reducing grey and white matter tissue loss, TUNEL+ cell death, microglia activation, reactive astrogliosis, and iNOS oxidative stress when compared to vehicle-treated littermates. This effect was dose-dependent, with 200 μg/g body weight as the optimal dose-regimen, and was maintained when curcumin treatment was delayed by 60 or 120 min post-HI. Cell proliferation measurements showed no changes between curcumin and HI alone, suggesting that the protective effects of curcumin on the neonatal brain following HI are most likely due to curcumin’s anti-inflammatory and antioxidant properties, as seen in the reduced glial and iNOS activity. In conclusion, this study suggests curcumin as a potent neuroprotective agent with potential for the treatment of HIE. The delayed application of curcumin further increases its clinical relevance.

## Introduction

Neonatal hypoxic-ischemic (HI) brain injury has an incidence of 1–3 per 1,000 live births (Rocha-Ferreira and Hristova, 2016), and results in almost 1 million neonatal deaths worldwide (Lawn et al., 2005; Rocha-Ferreira and Hristova, 2016). Approximately, 30% of HI cases will develop lifelong disabilities, including cerebral palsy, seizures, and cognitive impairments (Rocha-Ferreira and Hristova, 2016; Lundgren et al., 2018). The severity of such disabilities depends on the stage of gestation at which the HI event occurs and its duration (Sanders et al., 2010).

The pathology of HI brain damage is characterized by an initial primary energy loss phase, where oxygen and glucose deprivation in the cell causes a drop in the mitochondrial oxidative phosphorylation, resulting in reduced adenosine triphosphate (ATP) availability, triggering excitotoxicity (Rocha-Ferreira and Hristova, 2016), neurotoxicity (Sanders et al., 2010), and oxidative stress (Hope et al., 1984; Penrice et al., 1997). Early after the HI insult, damaged neuronal cells stimulate a pro-inflammatory immune response where activated microglia produce cytokines such as IL-1β and TNFα, proteases, and complement factors (Rocha-Ferreira and Hristova, 2016).

After successful resuscitation, a short latent/recovery period occurs. However, during reperfusion, the majority of the oxidative markers are produced, and in cases of an initial prolonged HI insult, the primary energy failure cannot be compensated, therefore, leading to a secondary energy drop (Rocha-Ferreira and Hristova, 2016). Inflammatory processes and continued excitotoxicity lead to an impaired equilibrium between pro- and anti-inflammatory cytokines, as well as significant damage of the mitochondria machinery (Peng and Greenamyre, 1998; Puka-Sundvall et al., 2000). This is associated with increased levels of hydrogen peroxide (H_2_O_2_) and nitrogen oxide (NO), overproduction of free radicals, and reactive oxygen species (ROS) which, together with the persistent inflammation, stimulate necrosis (Li et al., 1998), apoptosis (Johnston et al., 2002), and autophagy (Rocha-Ferreira and Hristova, 2016) cell death pathways.

Therapeutic hypothermia (TH) is the standard clinical treatment applied in moderate to severe injury; however, it does not guarantee total recovery of the treated neonates with effectiveness of only 55% of cases and the remaining infants still develop neurological deficits (Gluckman et al., 2005). Thus, further studies on improving TH success rate and finding therapeutic alternatives are urgently required.

Curcumin, the major phytochemical component of the plant *Curcuma longa*, is extracted from its rhizomes (turmeric), and regularly consumed in South Asian diets (Shishodia et al., 2005; Pescosolido et al., 2013). Except for turmeric usage as dietary spice and coloring agent, curcumin has been studied for its therapeutic role in several pathological conditions; its effects have been investigated in cancer (Naksuriya et al., 2014; Ahmad et al., 2016), inflammation (Kim et al., 2003; Sandur et al., 2007), infections, cardiovascular diseases (Nishiyama et al., 2005; Liu and Hong, 2006), fibrosis, and neurological disorders (Spagnuolo et al., 2016). Such pharmacological success relies on the phytochemical abilities of acting on many critical pathways, showing anti-inflammatory (Tham et al., 2010), anti-oxidant (Alexandrow et al., 2012), anti-microbial (Moghadamtousi et al., 2014), and anti-apoptotic (Yu et al., 2010; Jaisin et al., 2011) effects, as well as capability to promote stem cell differentiation (Gu et al., 2012; Mujoo et al., 2012; Chen et al., 2014). Effects of curcumin on mitochondrial function have also been reported (Bavarsad et al., 2018; He et al., 2018; Hsiao et al., 2018; Momekova et al., 2018; Naserzadeh et al., 2018) and furthermore, hormetic effects of curcumin are receiving increased attention (Moghaddam et al., 2018). Curcumin pleiotropic activity is due to its chemical structure, and specifically to the o-methoxy phenolic groups (Priyadarsini and Indira, 2014), which are central for chemical reactions. In fact, curcumin scavenging abilities on ROS and free radicals (Trujillo et al., 2014) seem to rely on this functional group, as well as its modulatory role on pro-inflammatory cytokines [tumor necrosis factor alpha (TNFα), interleukin (IL)1 (Alexandrow et al., 2012), and IL6 (Maheshwari et al., 2006)] and its inhibition of signal transducer and activator of transcription 3 (STAT3) phosphorylation (Maheshwari et al., 2006; Alexandrow et al., 2012). As STAT3 is crucial for astrocyte differentiation (Hong and Song, 2014), its inhibition can reduce reactive astrogliosis post-HI. Clinical trials have demonstrated remarkable safety profile and good tolerance for curcumin in humans at doses of 8 g/day (Dhillon et al., 2008). Moreover, the small molecular weight (368.385 g/mol), and dimensions allow it to cross the blood-barrier (BBB) (Priyadarsini and Indira, 2014), increase researchers interest for curcumin application in neurodegenerative disorders including traumatic brain injury (Wu et al., 2006), Alzheimer’s (Ray et al., 2011; Mutsuga et al., 2012), and Parkinson’s (Mythri et al., 2011) diseases.

The pathways on which curcumin acts, overlap with those activated after HI injury, including oxidative stress and inflammation. So far curcumin has been vaguely tested in neonatal HI injury in rats, either through oral administration (Cui et al., 2017) or through intraperitoneal single dose delivery of nanoparticle- or DMSO-dissolved curcumin (Joseph et al., 2018). Both studies report attenuation of damage; however, there is no systematic approach to dose response or long-term protection. In fact, Joseph et al. report no effect of the DMSO-dissolved curcumin suggesting that it is only the nanoparticle-loaded curcumin showing protection (Joseph et al., 2018). The aim of our study was to assess the short- and long term-effects of curcumin administered immediately after neonatal HI insult, to determine the lowest neuroprotective dose of the compound and whether delayed administration at 60 and 120 min post-HI would show neuroprotection. Our observations show that immediate or delayed administration of curcumin strongly protects the neonatal brain following HI insult. These protective effects are though not a consequence of changes in cell proliferation, but possibly related to alterations in STAT3 phosphorylation and prohibitin (PHB) protein levels. The STAT3 Y705 phosphorylation site is responsible for the transcription properties of STAT3 and previous results from our group showed bilateral upregulation and involvement of STAT3 Y705 in neonatal HI brain damage (Hristova et al., 2016). The STAT3 S727 phosphorylation site is downstream of extracellular signal-regulated kinase (ERK), respectively of RAS and is responsible for mitochondrial survival in cancer studies (Gough et al., 2009).

PHB is a pleiotropic multifaceted protein and an essential factor in mitochondrial homeostasis, biogenesis, degradation, and response to stress (Peng et al., 2015; Ande et al., 2017; Hernando-Rodríguez and Artal-Sanz, 2018). In mitochondria, PHB acts as a scaffold protein and is thus crucial for regulation of mitochondrial architecture, mitochondrial dynamics, morphology, and biogenesis, and furthermore stabilizes the mitochondrial genome (Merkwirth et al., 2012; Peng et al., 2015).

Our data demonstrate that the neuroprotective effects of curcumin are likely the consequence of changes in the levels of PHB and of STAT3 phosphorylation (Y705 and S727); thus affecting inflammation and mitochondrial dysfunction post-HI. Therefore, curcumin could be considered a potent candidate for neuroprotective treatment in neonatal HI brain damage.

## Materials and Methods

### HI Insult

All animal experiments and care protocols were carried out according to the UK Animals (Scientific Procedures) Act 1986 and approved by the Home Office (PPL70/8784). The ARRIVE guidelines were followed. All experiments involved postnatal day 7 C57/Bl6 mice (P7) bred in house.

The surgical procedures were performed as previously described (Hristova et al., 2010; Kendall et al., 2012; Lange et al., 2014; Rocha-Ferreira et al., 2015). Briefly, male and female P7 mice were anesthetized with isoflurane (5% induction and 1.5% maintenance). The left common carotid artery was permanently occluded with 8/0 polypropylene suture and the wound closed with tissue glue. The mice recovered at 36°C and were returned to the dam for 2 h. The pups were then placed in a hypoxia chamber and exposed to humidified 8% oxygen/92% nitrogen (3 L/min) at 36°C for 60 min, resulting in moderate to severe brain damage (Lange et al., 2014; Hristova et al., 2016).

The P7 rodent HI model, though slightly preterm, presents phenotypical similarities to the grey and white matter injury observed in humans, i.e., tissue loss, cell-death, microglia-mediated immune response, and astrogliosis as well as alteration in neurobehavioral performance (Vannucci and Vannucci, 1997).

### Pharmacological Treatment

Curcumin (LKT Laboratories, UK) was dissolved in 100% DMSO to concentrations of 20, 44, 100, 200, and 400 ug/ul. The animals were injected 20 min before, immediately after, or after a 60 or 120 min delay following a 60 min HI insult. The animals received a single intraperitoneal injection of 0.5 ul/g body weight (BW) resulting in doses of 10, 22, 50, 100, or 200 μg/g, respectively, based on previous studies (Shukla et al., 2008; Zhao et al., 2010).

### Tissue Sample Preparation

The animals were sacrificed at 48 h or at 21 days post-HI by intraperitoneal injection of pentobarbitone and perfused with 30 ml (for 48 h) or 90 ml (for 21 days) 4% paraformaldehyde in 0.1 M phosphate buffer (PB). The brains were removed, post fixed in 4% paraformaldehyde/0.1 M PB for 1 h at 4°C, and cryoprotected in 30% sucrose/PB solution for 24 h as previously described (Rocha-Ferreira et al., 2015; Hristova et al., 2016). The brains were then frozen on dry ice, cut on a cryostat into sequential 40 μm sections, and stored at −80°C until used.

### Immunohistochemistry and Histological Analysis

Five sections from each brain (400 μm apart) were rehydrated in distilled water and stained using immunohistochemistry as previously described (Möller et al., 1996). Briefly, the sections were incubated overnight with rat anti-CD11b αM integrin subunit (1:5,000, Serotec, UK), rabbit polyclonal anti-GFAP (1:6,000, DAKO, UK), rabbit anti-iNOS (1:500, Santa Cruz, USA), polyclonal rat anti-BrdU (1:400, Abcam, UK), or rabbit anti-myelin basic protein (MBP) (1:200, Abcam, Cambridge, UK) primary antibodies, for 1 h with biotinylated goat anti-rabbit or -rat (1:100, Vector, UK) secondary antibodies, followed by incubation with Avidin-Biotinylated horseradish peroxidase Complex (Vector, UK) and visualization with diaminobenzidine/H_2_O_2_ (Fisher Scientific, UK). The visualization for BrdU antibody required Co/Ni enhancement.

Five further sections from each brain with the same spacing were stained using Terminal transferase mediated d-UTP nick end labeling (TUNEL) (Roche, UK). The staining procedure followed the manufacturer protocol with Co/Ni enhancement.

Five more sections per brain with the same spacing were stained with Cresyl-Violet (Nissl).

To detect and identify the different types of proliferating cells following neonatal HI, we used double labeling for BrdU and: rabbit polyclonal anti-IBA1 (microglia, 1:2,000, Wako, Japan), rabbit polyclonal anti-GFAP (astroglia, 1:6,000, DAKO, UK), guinea pig polyclonal anti-NG2 (oligodendrocyte precursors, 1:400, Bill Stallcup, USA), or mouse monoclonal anti-NeuN (neurons, 1:25,000, Millipore, UK). The protocol was performed as previously described (Hristova et al., 2010). The sections were covered with VectaShield (Vector) and stored in the dark at 4°C before use. The number of BrdU positive and the double positive cells was counted in three fields at ×20 magnification. The percentage of the double positive cells as a fraction of the overall number was calculated and presented.

### AlphaM Score

Immunohistochemistry for αM integrin as an early microglial activation marker (Raivich et al., 1999; Hristova et al., 2010; Kendall et al., 2012; Lange et al., 2014; Rocha-Ferreira et al., 2015), was performed as previously described (Rocha-Ferreira et al., 2015; Hristova et al., 2016). Semi-quantitative scores were allocated to each brain region (cortex, pyriform cortex, hippocampus, striatum, thalamus, and external capsule) by two independent observers blinded to the treatment of the groups.

### TUNEL, BrdU, and iNOS

TUNEL positive cell death or iNOS and BrdU positive cells were assessed at 48 h following HI through bilateral counting of the number of positive cells in three different optical fields at ×20 magnification. Cortex, pyriform cortex, hippocampus, striatum, thalamus, and external capsule were assessed for TUNEL and BrdU. Due to the exclusive expression of iNOS in hippocampus, this marker was assessed only in that region. The counts were averaged per animal and per group.

### Optical Luminosity

The intensity of the GFAP or MBP staining in the tissue was assessed using optical luminosity values (Rocha-Ferreira et al., 2015; Hristova et al., 2016). Images for ipsilateral and contralateral sides were captured with a Sony AVT-Horn 3CCD color video camera (24 bit RGB, 760 × 570 pixel resolution) in three different optical fields in the regions of interest. We used Optimas 6.5 software to obtain the mean and standard deviation (SD) for optical luminosity values (OLV). SD was subtracted from the mean for each image and the resulting value was subtracted from the values acquired for the surrounding glass (Möller et al., 1996). OLVs for GFAP assessment were evaluated in cortex, pyriform cortex, hippocampus, striatum, thalamus and external capsule, while for MBP assessment OLVs were recorded in external capsule and striatum.

### Infarct Volume Measurement

The Cresyl-Violet stained sections were scanned and imported into Optimas 6.5 image analysis software. The areas of intact staining in the cortex, pyriform cortex, hippocampus, striatum, thalamus, and external capsule were outlined and bilaterally measured using Fiji Image J (NIH, USA). In the long-term assessment experiments the intact staining of the different regions as well as the whole hemisphere were outlined and bilaterally measured. The percentage tissue loss was then calculated by converting the measured injured and uninjured areas into square millimeters and then transformed to a volume through multiplication by 400 μm. The sum of these volumes was then used to calculate the percentage of surviving brain tissue as ipsilateral/contralateral × 100 (Kendall et al., 2006).

### Behavioral Assessment

The slipping test was performed at 21 days (P28) post-HI. It is a modification of the balance beam test (Luong et al., 2011) used for evaluation of motor balance and co-ordination. This test detects motor deficits resulting from central nervous system lesions, as well as aging and genetic and pharmacological interventions (Luong et al., 2011). The apparatus consists of a metal grid 50 cm in length, placed about 20 cm above a table top between the housing cage and a new clean cage. The starting point is the clean cage and the finish consisted of the housing nesting cage. The animals were allowed to walk freely on the grid for 1 min. The task was recorded, and the videos were reviewed by two team members blinded to the mouse groups. The number of missed steps was counted and presented as a percentage of the total number of steps for each animal. The results were then averaged per group.

At P7, the levels of testosterone in male and female mice do not differ (Clarkson and Herbison, 2016). However, at 21 days, testosterone levels between male and female mice are different and a lot of clinical and experimental evidence suggests important differences between males and females, with increased loss of male hippocampal volume after chronic postnatal hypoxia (Mayoral et al., 2009; Gobinath et al., 2017). Thus, the assessments for 21 days took gender into account.

### Western Blot Analysis

The animals were sacrificed at 1 h post HI by intraperitoneal injection of pentobarbitone and hippocampus was extracted from treated and un-treated brains and snaps frozen before homogenization in RIPA+ buffer (Sigma, UK) containing 10% protease inhibitor complex (Sigma, UK). The time point was chosen as coinciding with time of maximum expression of phosphorylated STAT3 Y705 (Hristova et al., 2016). Total protein was thereafter extracted from the homogenized hippocampal tissue as follows: the homogenized tissue was incubated with the RIPA+ buffer on a shaking platform on ice for 2 h, pipetting gently with regular intervals. Thereafter, the homogenates were centrifuged at 16,000 g at 4°C for 20 min and the supernatant containing the extracted protein collected. The protein extracts were re-constituted in 2 *×* Laemmli sample buffer (BioRad, UK) containing 5% β-mercaptoethanol (Sigma, UK) and boiled for 5 min at 100°C before separation by SDS-PAGE, using 4–20% Mini-Protean TGX protein gels (BioRad, UK), followed by Semi-Dry Western blotting analysis. Approximately, 5 μg of protein was loaded per lane and even transfer to nitrocellulose membranes (0.45 μm, BioRad, UK) was assessed using Ponceau S staining (Sigma, UK). The membranes were blocked for 1 h at room temperature (RT) in 5% bovine serum albumin (BSA, Sigma, UK) in Tris-buffered saline (TBS) with 0.001% Tween20 (TBS-T), followed by overnight incubation at 4°C with the following primary antibodies: anti-prohibitin (Abcam, UK), Phospho-Stat3 (Ser727) (Cell Signaling, UK and Abcam, UK), or Phospho-Stat3 (Tyr705) (Cell Signaling, UK and Abcam, UK). Thereafter, membranes were washed three times for 10 min in TBS-T, incubated for 1 h at RT with an HRP-labeled anti-rabbit IgG secondary antibody (BioRad, UK), followed by five TBS-T washes and one final TBS wash, and thereafter they were visualized using ECL (Amersham, UK) and the UVP BioDoc-ITTM System. HRP-conjugated anti-β-actin antibody (1/5,000 in TBS-T, Abcam, UK) was used for internal loading control and densitometry analysis was carried out using ImageJ.

### Statistics

Statistical significance was assessed through repeated testing using Mixed Linear Model with SPSS 23.0 and GraphPad Prism 7.0 software, treating region as the repeated measure. For each outcome six regions of the brain were examined. It is likely that with repeated measures such as the observations from a single subject are correlated, the first stage of the analysis included the observations from all the regions tested in a single mixed model with a random subject effect, to produce an estimate of the treatment effect and associated inference that accounts for the correlations in the data arising from the repeated measures. Further *post hoc* Student *t*-tests were carried out to assess evidence for subregional differences, *p* < 0.05. For comparison of more than two groups, we used two-way ANOVA with *post hoc* Tukey or Bonferroni tests to assess evidence for subregional differences, *p* < 0.05. If parametric analysis was inappropriate, the non-parametric Kruskal-Wallis test was used, followed by Bonferroni-corrected pairwise-contrasts to investigate differences between treatment-conditions. For each outcome, the main effect from the mixed linear model or the Kruskal-Wallis test is reported, followed by the results from the individual regional *t*-tests. In our data, a main effect is the effect of an independent variable (treatment) on a dependent variable (damage marker) averaged across the levels of any other independent variables (brain regions). All data are presented as Mean + SEM.

## Results

### Intraperitoneal Pre-treatment With Curcumin Increases the Glial Response Following HI-Insult in the Neonate

**Figure 1:**
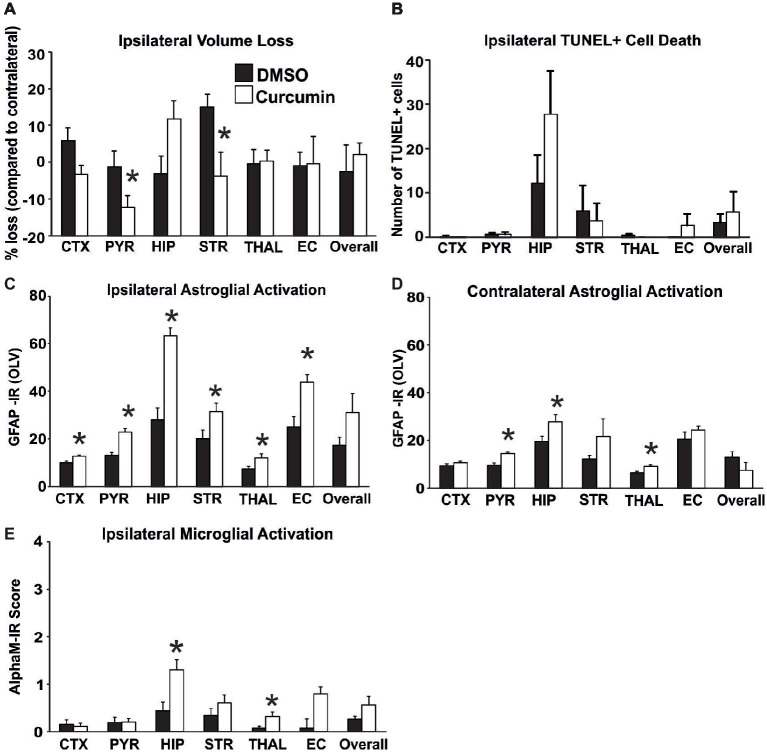
Intraperitoneal injection of curcumin (100 μg/g BW) in P7 mice 20 min before HI does not affect tissue damage and cell death, but significantly increases glial response. **(A)** Ipsilateral forebrain Nissl staining (Cresyl-Violet, at rostral parietal level) – Quantification of ipsilateral brain tissue volume loss of DMSO and curcumin pre-treated animals at 48 h following HI-insult. Curcumin pre-treatment (*n* = 12) did not affect volume loss compared to DMSO-treated littermates (*n* = 11) (Mixed Linear Model treating region as a repeated measure *p* = 0.156). However significant, individual decrease (*t*-test) was registered in pyriform cortex (*p* = 0.04) and striatum (*p* = 0.02). **(B)** The number of TUNEL+ dying cells (per 20x eye-field) at 48 h following HI, was not affected in the curcumin pre-treated group compared to DMSO-treated littermates (Mixed Linear Model treating region as a repeated measure *p* = 0.053). **(C,D)** GFAP immunoreactivity at 48 h – quantification of the ipsilateral **(C)** and contralateral **(D)** side in optical luminosity values (OLV, Mean + SEM). Note the increased levels of GFAP immunoreactivity in the curcumin pre-treated animals, with significant, individual increase (*t*-test) in ipsilateral cortex (*p* = 0.002), pyriform cortex (*p* = 0.0001), hippocampus (*p* = 3×10^−5^), striatum (*p* = 0.038), thalamus (*p* = 0.026), and external capsule (*p* = 0.003), and contralateral pyriform cortex (*p* = 0.0002), hippocampus (*p* = 0.04), and thalamus (*p* = 0.013). Mixed Linear Model treating region as a repeated measure revealed *p* = 0.0001 for ipsilateral and *p* = 0.007 for contralateral side, respectively. **(E)** Activation of αM+ microglia – ipsilateral αM microglial activation score (Mean + SEM). Pre-treatment with 100 μg/g BW curcumin increased αM+ microglial activation with significant, individual increase (*t*-test) in hippocampus (*p* = 0.008) and thalamus (*p* = 0.03). Mixed Linear Model treating region as a repeated measure revealed *p* = 0.019. (*p < 0.05). Abbreviations: CTX, cerebral cortex; EC, external capsule; HIP, hippocampus; PYR, pyriform cortex; STR, striatum; THAL, thalamus.

To determine the biological impact of pre-treatment with curcumin, the animals were injected intraperitoneally with 100 μg/g BW curcumin and the effect on brain volume loss, TUNEL+ cell death, and on reactive astrogliosis and microglial activation at 48 h were examined following the HI-insult. As shown in Figure 1, curcumin pre-treatment had no main effect on brain volume loss (Figure 1A) and TUNEL+ cell death (Figure 1B). However, individual significant attenuation of brain volume loss (*t*-test) was observed in pyriform cortex and striatum (Figure 1A). Interestingly, an increase of tissue loss, although not significant, was observed in the hippocampus of curcumin treated animals. Pre-treatment with 100 μg/g BW of curcumin significantly increased HI-induced and predominantly ipsilateral reactive astrogliosis and microglial activation (Figures 1C–E). Compared to DMSO-treated littermates, the curcumin pre-treated animals revealed more GFAP-immunoreactivity (Figures 1C,D). Assessment across the different forebrain regions through Mixed Linear Model treating region as a repeated measure revealed a clear increase on the ipsilateral, and milder increase on the contralateral side (*p* = 0.0001 and *p* = 0.007, respectively), with individual significant increase of 20–50% in all studied ipsilateral regions, and of 30–40% in contralateral pyriform cortex, hippocampus and striatum (*p* < 0.05 in *t*-test). Curcumin pre-treatment also increased microglial activation score (Figure 1E) based on the αM integrin immunoreactivity. Regional assessment revealed an increase in activation score in the curcumin pre-treated group (Mixed Linear Model treating region as a repeated measure *p* = 0.019) with significant increase of 30–50% in hippocampus and thalamus (Figure 1E).

### Immediate Post-treatment With Curcumin Reduces Brain Damage Following Neonatal HI-Insult

**Figure 2:**
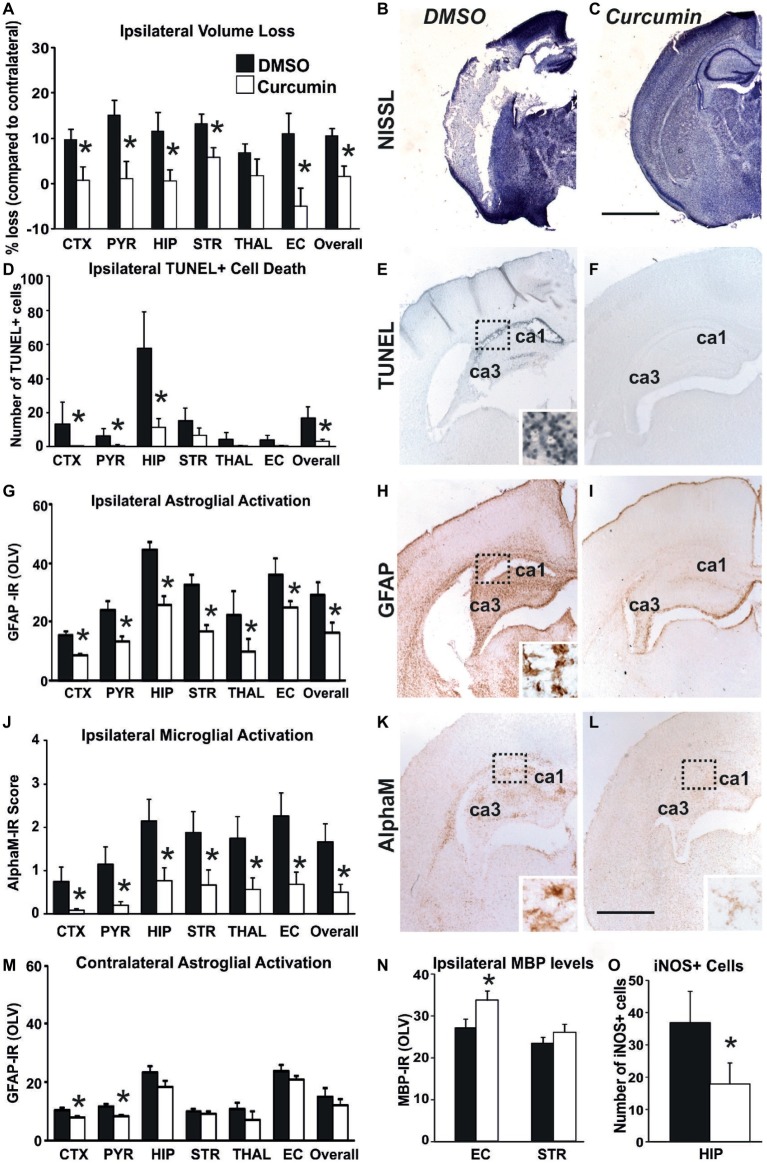
Intraperitoneal injection of curcumin (200 μg/g BW) in P7 mice immediately post-HI significantly reduces tissue damage, cell death and glial response. **(A–C)** Ipsilateral forebrain Nissl staining (Cresyl-Violet, at rostral parietal level) - Quantification of ipsilateral brain tissue volume loss **(A)** at 48 h following HI-insult of DMSO **(B)** and curcumin **(C)** treated animals at 48 h following HI-insult. Curcumin treatment (*n* = 11) reduced volume loss compared to DMSO-treated littermates (*n* = 11) with significant, individual decrease (*t*-test) in cortex (*p* = 0.026), pyriform cortex (*p* = 0.011), hippocampus (*p* = 0.038), striatum (*p* = 0.020), external capsule (*p* = 0.015) and overall volume loss (*p* = 0.003). Mixed Linear Model treating region as a repeated measure revealed *p* = 0.001. **(D–F)** TUNEL+ staining of dying brain cells with fragmented DNA at 48 h following HI-insult – Quantification **(D)** (number of TUNEL+ cells per 20× eye-field, Mean + SEM) and histochemical overview of the ipsilateral forebrain in DMSO **(E)** and curcumin **(F)** treated animals. Note the typical pyknotic nuclear morphology of the TUNEL+ cells observed in the DMSO group (**E**- insert, hippocampus) and the lack of such cells in the curcumin group **(F)**. Curcumin treatment reduced TUNEL+ cell death across all 6 examined forebrain regions, with significant, individual decrease (*t*-test) in cortex (*p* = 0.04), pyriform cortex (*p* = 0.049), hippocampus (*p* = 0.046), and overall (*p* = 0.048). Mixed Linear Model treating region as a repeated measure revealed *p* = 0.036. **(G–I,M)** GFAP immunoreactivity at 48 h - Quantification of the ipsilateral **(D)** and contralateral, non-occluded side **(M)** in optical luminosity values (OLV, Mean + SEM), and low magnification ipsilateral overview in DMSO **(H)** and curcumin **(I)** treated animals. The insert in **H** shows higher magnification of the dotted region in rostro-parietal cortex. Note the reduced levels of GFAP immunoreactivity in the curcumin treated animals, with significant, individual decrease (*t*-test) in ipsilateral cortex (*p* = 4 × 10^−5^), pyriform cortex (*p* = 0.004), hippocampus (*p* = 0.049), striatum (*p* = 0.001), thalamus (*p* = 0.001), external capsule (*p* = 0.009) and overall (*p* = 0.048) in **G**, and in contralateral cortex (*p* = 0.01) and pyriform cortex (*p* = 0.0004) in **M**. Mixed Linear Model treating region as a repeated measure revealed *p* = 0.005 for the ipsilateral, and *p* = 0.0001 for the contralateral side. **(J–L)** Activation of αM+ microglia – Ipsilateral αM microglial activation score (**J**, Mean + SEM) and low magnification ipsilateral overview in DMSO **(K)** and curcumin **(L)** treated animals. Note the strong microglial activation in DMSO-treated animals with αM+ cells showing phagocytic morphology at high magnification (K-insert, hippocampus), compared to the curcumin treated brains exhibiting a ramified phenotype (L-insert). Curcumin treatment reduced αM+ microglial activation across all six examined forebrain regions, with significant, individual decrease (*t*-test) in cortex (*p* = 0.03), pyriform cortex (*p* = 0.01), hippocampus (*p* = 0.03), striatum (*p* = 0.04), thalamus (*p* = 0.04), external capsule (*p* = 0.01) and overall (*p* = 0.02). Mixed Linear Model treating region as a repeated measure *p* = 0.019. DMSO (*n* = 11) and curcumin (*n* = 11) in all assessments. **(N)** MBP immunoreactivity at 48 h - Quantification of the ipsilateral external capsule and striatum in optical luminosity values (OLV, Mean + SEM). Note the increased levels of MBP immunoreactivity in the curcumin treated animals, with significant, individual increase (*t*-test) in ipsilateral external capsule (*p* = 0.045). Mixed Linear Model treating region as a repeated measure *p* = 0.056. **(O)** iNOS immunoreactivity at 48 h – Quantification of the number of iNOS+ cells in ipsilateral hippocampus (number of iNOS+ cells per 20× eye-field, Mean + SEM). Note the reduced number of iNOS+ cells (*t*-test) in the curcumin treated group compared to DMSO-treated littermates (*p* = 0.041). (*p < 0.05). Abbreviations: CTX, cerebral cortex; EC, external capsule; HIP, hippocampus; PYR, pyriform cortex; STR, striatum; THAL, thalamus. Scale bars: **(B,C)** = 2,000 μm; **(E,F,H,I,K,L)** = 1,000 μm; inserts = 62 μm.

Compared to DMSO-treated animals, intraperitoneal injection of 200 μg/g BW curcumin straight after the HI-insult significantly reduced brain damage markers (tissue loss, TUNEL+ cell death, reactive astrogliosis, and microglial activation) at 48 h post-HI. As shown in Figures 2A–C, curcumin post-treatment markedly decreased ipsilateral forebrain tissue loss. Regional assessment presented in Figure 2A revealed strong decrease across the different forebrain regions (Mixed Linear Model treating region as a repeated measure, *p* = 0.001). Treatment with curcumin significantly reduced tissue loss in relation to DMSO-treated littermates by 50–90% in cortex, pyriform cortex, hippocampus, striatum, external capsule, and overall forebrain area (*p* < 0.05 in *t*-test). Figure 2B shows large infarct in cortex and hippocampus of the DMSO-treated animal and its sparing in the curcumin-treated littermate (Figure 2C).

A similar effect of the curcumin post-treatment was also observed for TUNEL+ cell death (Figures 2D–F). Figure 2D shows that curcumin treatment straight after 60 min HI significantly reduced the number of TUNEL+ cells compared to DMSO-treated littermates (Mixed Linear Model treating region as a repeated measure *p* = 0.036), with individual significant decrease of 70–90% in cortex, pyriform cortex, hippocampus, and overall (*p* < 0.05 in *t*-test). The TUNEL+ cells displayed the typical pyknotic nuclear morphology (Figure 2E-insert, ipsilateral hippocampus DMSO).

In addition to cell death and brain tissue loss, curcumin post-treatment also decreased HI-induced and predominantly ipsilateral reactive astrogliosis and microglial activation. Compared to DMSO-treated animals, their curcumin-treated littermates revealed less GFAP immunoreactivity (Figures 2G,M) with substantially reduced amount of GFAP+ astroglial processes (Figures 2H,I). Assessment across the different forebrain regions through Mixed Linear Model treating region as a repeated measure revealed a clear decrease on the ipsilateral, and milder reduction on the contralateral side (main effect *p* = 0.005 and *p* = 0.0001, respectively) with individual significant decrease of 30–50% in all six ipsilateral regions and overall, and of 30% in contralateral cortex and pyriform cortex (*p* < 0.05, *t*-test).

Curcumin post-treatment had a similar effect on microglia activation score (Figure 2J) based on αM integrin immunoreactivity (Figures 2K,L). Regional assessment shown in Figure 2J revealed a reduction in activation score in the curcumin-treated group (Mixed Linear Model treating region as a repeated measure, main effect *p* = 0.019), with significant decrease of 70–90% in all six individual ipsilateral brain regions (*p* < 0.05 in *t*-test).

Compared to DMSO-treated animals, curcumin post-treated littermates had higher levels of myelination assessed through MBP immunoreactivity (Figure 2N), with individual significant increase of 20% in external capsule (*p* < 0.05, *t*-test).

Curcumin post-treatment also reduced the levels of oxidative stress assessed through iNOS immunoreactivity, compared to DMSO-treated littermates, with individual significant decrease of 50% in hippocampus (Figure 2O, *p* < 0.05, *t*-test).

### Immediate Post-treatment With Curcumin Reduces Ipsilateral Volume Loss in Males, Females and Combined (Males + Females), Decreases Myelin Loss in Males, but Does Not Provide Functional Protection Assessed Through the Slipping Test

To evaluate the long-term effects of curcumin following immediate injection of 200 μg/g post HI, the levels of tissue loss were assessed through Nissl staining, the degree of myelination was evaluated through MBP immunoreactivity, and motor balance and co-ordination were assessed through the slipping test (Rocha-Ferreira et al., 2018) at day 21 post HI (P28).

**Figure 3:**
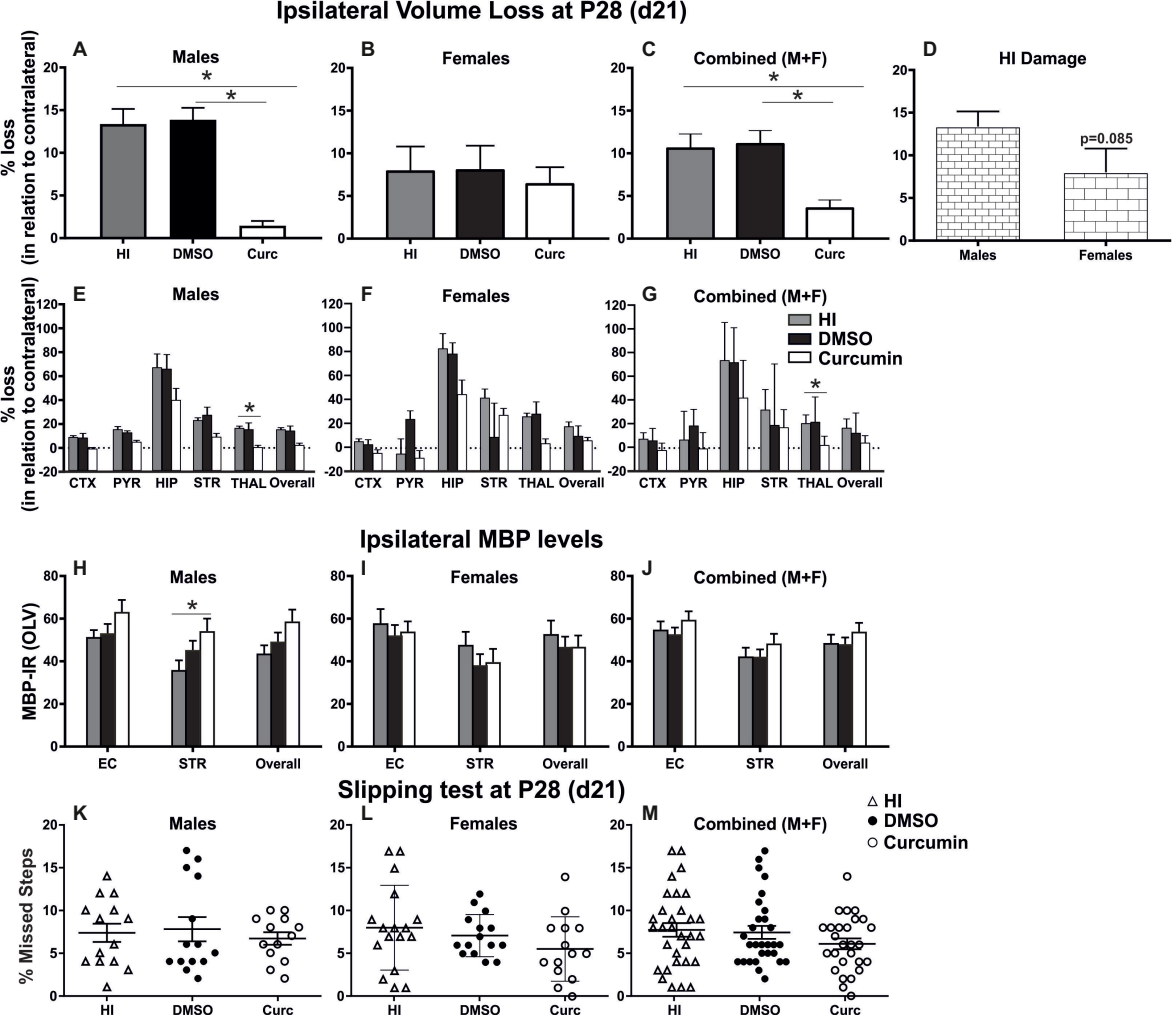
Intraperitoneal injection of curcumin in P7 mice post-HI reduces ipsilateral volume loss in males, females and combined (males + females), decreases myelin loss in males, but does not provide functional protection assessed through slipping test. **(A)** Intraperitoneal injection of 200 μg/g curcumin straight after HI decreased tissue loss at 21 days post HI (P28) in males compared to DMSO-treated littermates and untreated HI controls (curcumin *n* = 11, DMSO *n* = 8, HI *n* = 9; curcumin vs. HI *p* = 0.0017, curcumin vs. DMSO *p* = 0.0003). **(B)** Curcumin treatment at 200 μg/g straight after HI did not affect tissue loss at 21 days post HI in females compared to DMSO-treated littermates or to untreated HI controls. (curcumin *n* = 8, DMSO *n* = 7, HI *n* = 8). **(C)** The levels of tissue loss in the curcumin treated animals decreased compared to DMSO-treated littermates and untreated HI controls (curcumin *n* = 19, DMSO *n* = 15, HI *n* = 17; curcumin vs. HI *p* = 0.0024, curcumin vs. DMSO *p* = 0.0014). **(D)** The level of tissue loss observed in females was lower compared to the males, however the differences did not reach significant values (*p* = 0.085). This suggests higher susceptibility of the males to HI insult. **(E–G)** Subregional assessment of tissue loss at 21 days post HI in males (**E**, curcumin *n* = 11, DMSO *n* = 8, HI *n* = 9), females (**F,** curcumin *n* = 8, DMSO *n* = 7, HI *n* = 8) and combined (males + females) (**G**, curcumin *n* = 19, DMSO *n* = 15, HI *n* = 17). Curcumin treatment with 200 μg/g immediately post HI resulted in a reduction of subregional tissue loss in all studied regions in males **(E)** and combined (males + females) **(G)** with significant differences observed in thalamus (males *p* = 0.0292, combined (males + females) *p* = 0.0242). **(F)** Curcumin treatment had no effect on subregional differences in tissue loss in females. **(H–J)** MBP immunoreactivity at 21 days post HI (P28). Quantification of the ipsilateral external capsule, striatum, and overall in optical luminosity values (OLV, Mean + SEM). **(H)** In males curcumin treatment with 200 μg/g immediately post HI resulted in increased levels of MBP immunoreactivity compared to untreated HI controls (curcumin *n* = 11, DMSO *n* = 8, HI *n* = 9), with significant differences in striatum (Two-way ANOVA with *post hoc* Tukey’s test, *p* = 0.042). In females (**I**, curcumin *n* = 8, DMSO *n* = 7, HI *n* = 8) and combined (males + females) (**J**, curcumin *n* = 19, DMSO *n* = 15, HI *n* = 17), immediate treatment with 200 μg/g curcumin had no effect on MBP immunoreactivity. **(K–M)** Curcumin treatment with 200 μg/g immediately post HI did not affect the number of missed steps (slipping test) at 21 days (P28) post HI in males (**K**, curcumin *n* = 13, DMSO *n* = 14, HI *n* = 14), females (**L**, curcumin *n* = 14, DMSO *n* = 15, HI *n* = 17) and combined (males + females) (**M**, curcumin *n* = 27, DMSO *n* = 29, HI *n* = 31). (*p < 0.05). Abbreviations: CTX, cerebral cortex; EC – external capsule; HIP – hippocampus; PYR, pyriform cortex; STR, striatum; THAL, thalamus.

Intraperitoneal injection of 200 μg/g curcumin straight after HI decreased tissue loss in males at 21 days post HI compared to DMSO-treated littermates and untreated HI controls (Figure 3A). Immediate curcumin treatment had no effect on tissue loss in females compared to DMSO treated or untreated HI controls (Figure 3B). The levels of tissue loss in females were lower compared to males (Figure 3D). Although the differences did not reach significant values, the lower level of tissue loss in females suggests increased susceptibility of males to the insult. The levels of tissue loss in the curcumin group were significantly lower compared to DMSO-treated littermates and untreated HI controls (Figure 3C). Subregional assessment of tissue loss at 21 days post HI in males (Figure 3E) and combined (males + females) (Figure 3G) suggested that immediate treatment with curcumin at 200 μg/g immediately post HI reduced tissue damage in all studied regions with significant decrease in thalamus. Curcumin treatment did not affect subregional differences in tissue loss in females (Figure 3F).

Compared to untreated male HI control animals, curcumin post-treated littermates had an increase in myelination assessed through MBP immunoreactivity (Figure 3H) of 23% in striatum (*p* < 0.05, two-way ANOVA). Immediate post-HI treatment with 200 μg/g curcumin did not affect myelin loss in females (Figure 3I) or combined (males + females) (Figure 3J).

Assessment of motor balance and co-ordination through slipping test at 21 days post HI of animals treated immediately after the insult with 200 μg/g curcumin, DMSO, or HI controls showed slightly decreased number of missed steps in curcumin-treated males (Figure 3K), females (Figure 3L) and combined (males + females) (Figure 3M); however, the differences did not reach significance (Kruskal-Wallis test).

### Immediate Post-treatment With Curcumin Reduces Brain Damage Following Neonatal HI-Insult in a Dose-Dependent Manner

**Figure 4:**
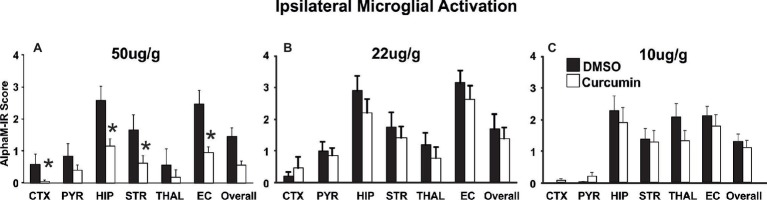
Intraperitoneal injection of curcumin in P7 mice post-HI significantly reduces ipsilateral αM microglial activation score in a dose dependent manner. **(A)** Intraperitoneal injection of 50 μg/g BW curcumin decreased αM+ microglial activation compared to DMSO-treated littermates, with significant, individual decrease (*t*-test) in cortex (*p* = 0.038), hippocampus (*p* = 0.035), striatum (*p* = 0.035) and external capsule (*p* = 0.046). Mixed Linear Model treating region as a repeated measure revealed *p* = 0.035. **(B,C)** Intraperitoneal curcumin injection of 22 μg/g **(B)** or 10 μg/g **(C)** did not have an effect on αM+ microglial activation compared to DMSO-treated littermates. (**A**, curcumin 50 μg/g *n* = 8, DMSO *n* = 6, **B**, curcumin 22 μg/g *n* = 12, DMSO *n* = 10, **C**, curcumin 10 μg/g *n* = 11, DMSO *n* = 11). (*p < 0.05) Abbreviations: CTX, cerebral cortex; EC, external capsule; HIP, hippocampus; PYR, pyriform cortex; STR, striatum; THAL, thalamus.

Curcumin dose of 200 μg/g BW resulted in significant neuroprotection (Figure 2). To determine whether a lower dose of curcumin post-treatment immediately after the HI-insult would provide neuroprotective effects, three groups of animals were injected intraperitoneally with doses of 50, 22, and 10 μg/g BW curcumin in 0.5 μl/g BW DMSO. The control littermates in each group received the same volume of DMSO only. Brain damage markers (tissue loss, TUNEL+ cell death, reactive astrogliosis, and microglial activation) were assessed at 48 h post-HI. As shown in Figure 4, the dose of 50 μg/g (Figure 4A) significantly reduced the levels of microglial activation (Mixed Linear Model treating region as a repeated measure *p* = 0.035), with significant individual decrease of 50–65% in cortex, hippocampus, striatum, and external capsule (*p* < 0.05, *t*-test). Similarly, the dose of 50 μg/g BW curcumin significantly reduced the levels of ipsilateral tissue loss (Mixed Linear Model treating region as a repeated measure *p* = 0.0001), with significant individual decrease of 75–90% in cortex, hippocampus, and overall (Supplementary Figure S1A, *p* < 0.05, *t*-test). The levels of TUNEL+ cell death were also reduced by the dose of 50 μg/g BW curcumin (Mixed Linear Model treating region as a repeated measure *p* = 0.016) with significant individual reduction of 75–90% in hippocampus and external capsule (Supplementary Figure S1D, *p* < 0.05, *t*-test). The dose of 50 μg/g markedly reduced also the levels of ipsilateral reactive astrogliosis (Mixed Linear Model treating region as a repeated measure *p* = 0.014), with significant individual reduction of 44–55% in pyriform cortex, hippocampus, thalamus, and external capsule (Supplementary Figure S1G, *p* < 0.05, *t*-test). In a similar pattern, the dose of 50 μg/g significantly reduced contralateral astroglial activation (Mixed Linear Model treating region as a repeated measure *p* = 0.005), with significant individual decrease in pyriform cortex (*p* < 0.05, Supplementary Figure S1J). The doses of 22 and 10 μg/g BW curcumin did not have an effect on microglial activation (Figures 4B,C), ipsilateral volume loss, TUNEL+ cell death or reactive astrogliosis (Supplementary Figures S1B,C,E,F,H,I,K,L).

### Delayed Post-treatment With Curcumin at 60 and 120 min Post HI Reduces Brain Damage Following Neonatal HI-Insult

**Figure 5:**
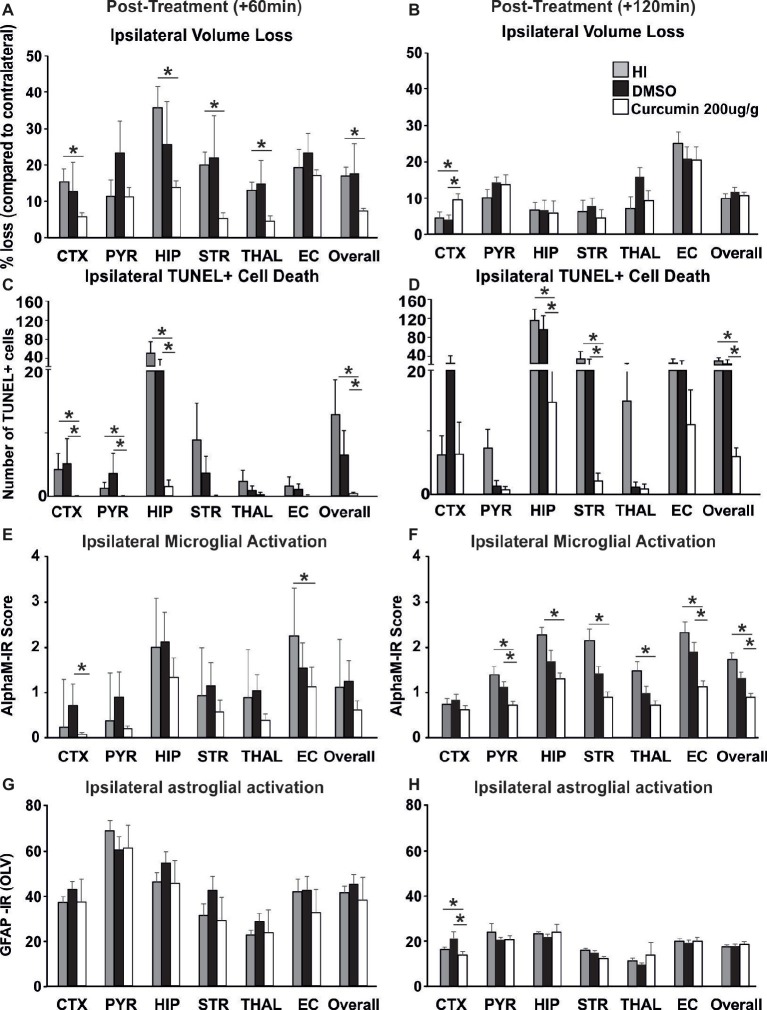
Intraperitoneal injection of curcumin (200 μg/g BW) in P7 mice at 60 min or 120 min post-HI significantly reduces tissue damage, cell death and glial response. **(A)** Curcumin treatment at 60 min post HI (*n* = 8) reduced volume loss compared to HI controls (*n* = 10) with significant, individual decrease (Kruskal-Wallis test with Bonferroni correction) in cortex (*p* = 0.036), hippocampus (*p* = 0.007), striatum (*p* = 0.003), thalamus (*p* = 0.005), and overall volume loss (*p* = 0.002). However, no main effect of the treatment was observed. No differences were registered between curcumin and DMSO (*n* = 7) treated littermates. **(B)** Administration of curcumin at 120 min post HI (*n* = 7) surprisingly increased the level of tissue loss however no significant main effect of treatment was observed. Pairwise comparison (one-way ANOVA with Bonferroni correction) revealed significant increase of volume loss in cortex in curcumin treated compared to HI (*n* = 7, *p* = 0.05) and to DMSO-treated littermates (*n* = 7, *p* = 0.017). **(C)** Curcumin treatment at 60 min post HI reduced TUNEL+ cell death compared to HI- and DMSO-treated littermates (Kruskal-Wallis test). Pairwise comparison (Bonferroni correction) revealed significant decrease of TUNEL+ cell death in curcumin treated compared to HI animals in cortex (*p* = 0.064), pyriform cortex (*p* = 0.064), hippocampus (*p* = 0.003) and overall (*p* = 0.001). Pairwise comparison (Bonferroni correction) revealed significant decrease of TUNEL+ cell death in curcumin compared to DMSO-treated animals in cortex (*p* = 0.003), pyriform cortex (*p* = 0.013), hippocampus (*p* = 0.042), and overall (*p* = 0.012). **(D)** Administration of curcumin at 120 min post HI reduced TUNEL+ cell death compared to HI- and DMSO-treated littermates (tone-way ANOVA, main effect *p* = 0.001). Pairwise comparison (Bonferroni correction) revealed significant decrease of TUNEL+ cell death in curcumin treated compared to HI animals in hippocampus (*p* = 0.002), striatum (*p* = 0.02), and overall (*p* = 0.001). Pairwise comparison (Bonferroni correction) revealed significant decrease of TUNEL+ cell death in curcumin compared to DMSO-treated littermates in hippocampus (*p* = 0.012), striatum (*p* = 0.022) and overall (*p* = 0.002). **(E)** Administration of curcumin at 60 min post HI reduced microglial activation compared to DMSO-treated littermates and HI controls, although the main effect of treatment did not reach significance. However, compared to HI controls, curcumin treatment reduced microglial activation with significant, individual decrease (Kruskal-Wallis test with Bonferroni correction) in external capsule (*p* = 0.042). Compared to DMSO-treated littermates curcumin treatment at 60 min post-HI reduced microglial activation with significant, individual decrease (Kruskal-Wallis test with Bonferroni correction) in cortex (*p* = 0.028). **(F)** Administration of curcumin at 120 min post HI reduced microglial activation compared to HI- and DMSO-treated littermates (Kruskal-Wallis test, main effect *p* = 0.002). Pairwise comparison (Bonferroni correction) revealed significant decrease of microglia activation in curcumin treated compared to HI animals in pyriform cortex (*p* = 0.03), hippocampus (*p* = 0.002), striatum (*p* = 0.002), thalamus (*p* = 0.01), external capsule (*p* = 0.006) and overall (*p* = 0.004). Pairwise comparison (Bonferroni correction) revealed significant decrease of microglia activation in curcumin compared to DMSO-treated littermates in pyriform cortex (*p* = 0.018), external capsule (*p* = 0.022), and overall (*p* = 0.016). **(G)** Curcumin treatment at 60 min post HI did not affect ipsilateral reactive astrogliosis. **(H)** Administration of curcumin at 120 min post HI reduced ipsilateral reactive astrogliosis (Kruskal-Wallis test with Bonferroni correction) in cortex compared to HI (*p* = 0.049) and DMSO-treated littermates (*p* = 0.006), however no main effect of the treatment was observed. (*p < 0.05). Abbreviations: CTX, cerebral cortex; EC – external capsule; HIP, hippocampus; PYR, pyriform cortex; STR, striatum; THAL, thalamus.

To investigate whether delayed application of curcumin would provide similar neuroprotection to the one achieved through immediate post-HI treatment, 200 μg/g BW curcumin was applied at 60 and 120 min following a neonatal HI insult and the brain damage markers were assessed at 48 h post HI. Treatment at 60 min reduced volume loss by 60–70% in cortex, hippocampus, striatum, thalamus, and overall compared to HI controls (Figure 5A, *p* < 0.05, Kruskal-Wallis test with Bonferroni correction), but had no effect compared to DMSO-treated littermates and no main effect of the treatment was observed. Application of curcumin at 60 min post HI significantly reduced TUNEL+ cell death compared to HI- and DMSO-treated littermates (Figure 5C, *p* < 0.05, Kruskal-Wallis test). Pairwise comparison between the curcumin treated and HI groups revealed significant 80–90% decrease of the number of TUNEL+ cells in cortex, pyriform cortex, hippocampus and overall (Figure 5C, *p* < 0.05, Kruskal-Wallis test with Bonferroni correction). Compared to DMSO-treated littermates curcumin treatment at 60 min post HI significantly reduced TUNEL+ cell death by 80–90% in cortex, pyriform cortex, hippocampus and overall (Figure 5C, *p* < 0.05, Kruskal-Wallis test with Bonferroni correction). Administration of curcumin at 60 min post HI reduced microglial activation compared to DMSO-treated littermates and HI controls, although the main effect of treatment did not reach significance (Figure 5E). Curcumin treatment at 60 min post HI reduced microglial activation compared to HI controls with significant, individual decrease of 30% in external capsule (Figure 5E, *p* < 0.05, Kruskal-Wallis test with Bonferroni correction). Compared to DMSO-treated littermates curcumin treatment at 60 min post-HI reduced microglial activation with significant, individual decrease of 90% in cortex (Figure 5E, *p* < 0.05, Kruskal-Wallis test with Bonferroni correction). Curcumin application at 60 min post HI did not affect ipsilateral astroglial activation (Figure 5G).

Application of curcumin at 120 min post HI surprisingly increased the level of tissue loss but there was no significant main effect of treatment (*p* > 0.05, one-way ANOVA). However pairwise comparison revealed significant increase of 50% in volume loss in the cortex of curcumin treated compared to HI and to DMSO-treated littermates (Figure 5B, *p* < 0.05, one-way ANOVA with Bonferroni correction). Delayed administration of curcumin at 120 min post HI significantly reduced the number of TUNEL+ cells compared to HI- and DMSO-treated littermates (Figure 5D, *p* < 0.05, one-way ANOVA). Pairwise comparison revealed significant 60–90% decrease of TUNEL+ cell death in curcumin treated compared to HI animals in hippocampus, striatum and overall (Figure 5D, *p* < 0.05, one-way ANOVA with Bonferroni correction). Pairwise comparison between curcumin and DMSO-treated animals revealed significant 50–85% decrease of TUNEL+ cell death in hippocampus, striatum and overall (Figure 5D, *p* < 0.05, one-way ANOVA with Bonferroni correction). Administration of curcumin at 120 min post HI reduced microglial activation compared to HI- and DMSO-treated littermates (Figure 5F, *p* < 0.05, Kruskal-Wallis test). Pairwise comparison between curcumin treated and HI animals revealed significant 40–60% decrease of microglial activation in pyriform cortex, hippocampus, striatum, thalamus, external capsule and overall (Figure 5F, *p* < 0.05, Kruskal-Wallis test with Bonferroni correction). Pairwise comparison revealed significant 50% decrease of microglial activation in curcumin compared to DMSO-treated littermates in pyriform cortex, external capsule and overall (Figure 5F, *p* < 0.05, Kruskal-Wallis test with Bonferroni correction). Administration of curcumin at 120 min post HI reduced ipsilateral reactive astrogliosis in cortex by 18% compared to HI and by 38% compared to DMSO-treated littermates (Figure 5H, *p* < 0.05, Kruskal-Wallis test with Bonferroni correction); however, no main effect of the treatment was observed.

### Post-treatment With Curcumin Immediately Post HI Has No Effect on the Levels of Cellular Proliferation at 48 h

**Figure 6:**
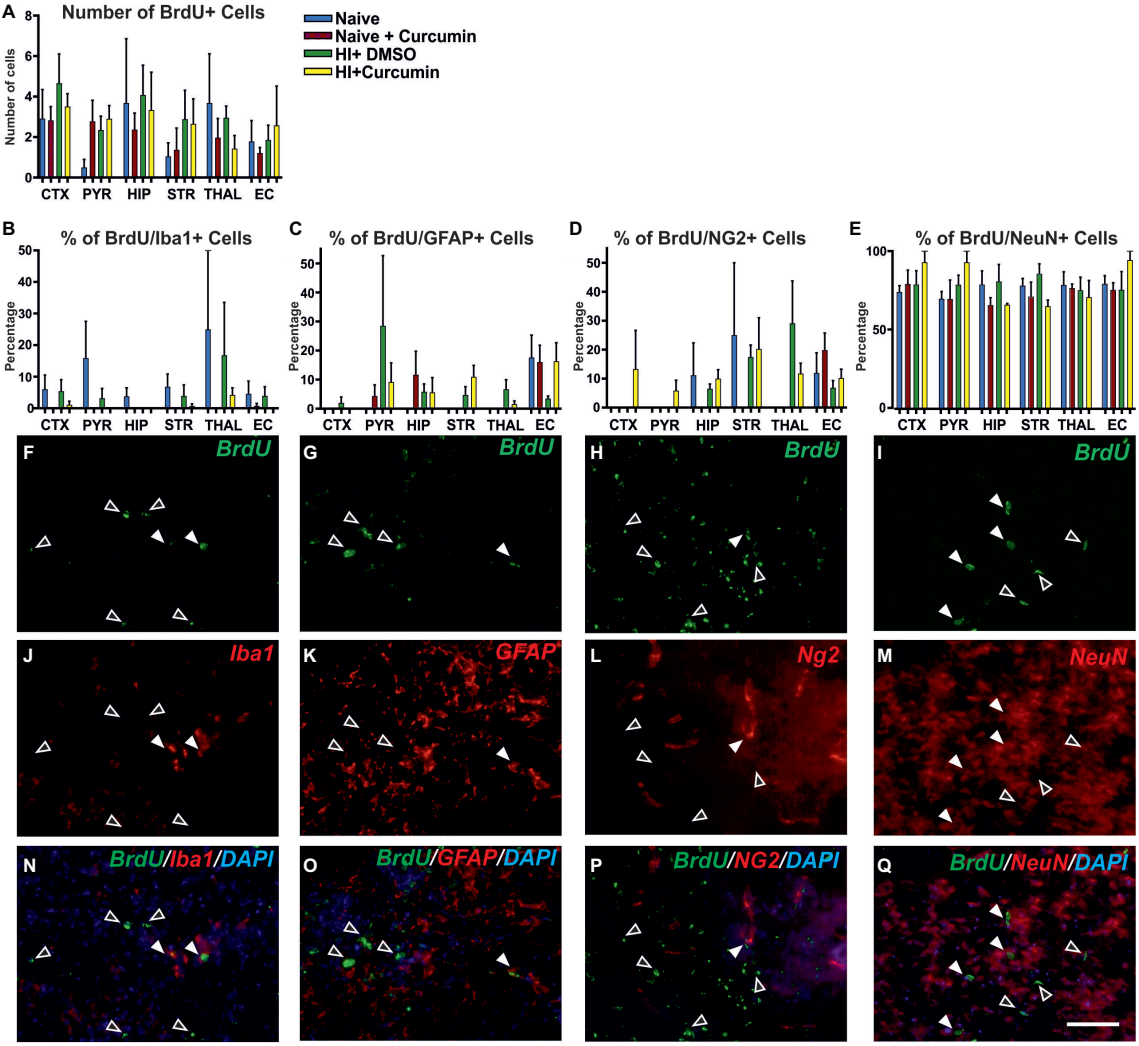
Intraperitoneal injection of curcumin (200 μg/g BW) in P7 mice immediately post-HI has no effect on the levels of proliferation at 48 h. **(A)** Assessment of the number of BrdU+ cells in P7 naïve untreated animals or naïve and HI animals treated with curcumin or DMSO did not show differences between the groups in any of the regions. (naïve *n* = 4, naïve + curcumin *n* = 5, HI + DMSO *n* = 7, HI + curcumin *n* = 8). **(B)** Curcumin treatment in naïve or HI animals immediately after insult did not affect the percentage of IBA1 and BrdU double positive over BrdU+ cells per region and group. The percentage of proliferating IBA1+ cells was low (10–20% of the BrdU+ cells). **(C)** The percentage of GFAP and BrdU double positive over BrdU+ cells per region and group was not affected by curcumin treatment in naïve or HI animals. The percentage of proliferating GFAP+ cells was low (10–20% of the BrdU+ cells). **(D)** The percentage of NG2 and BrdU double positive over BrdU+ cells per region and group was not affected by curcumin treatment in naïve or HI animals. The percentage of proliferating NG2+ cells was low (10–20% of the BrdU+ cells). **(E)** The percentage of NeuN and BrdU double positive over BrdU+ cells per region and group was not affected by curcumin treatment in naïve or HI animals. The percentage of proliferating NeuN+ cells comprised 80–90% of the BrdU+ cells and was the highest of all four studied cell types. **(F,J,N)** Immunofluorescence for rat polyclonal anti-BrdU **(F,N)** in green superimposed on the rabbit polyclonal anti-IBA1 **(J,N)** in red, and nuclear DAPI fluorescence in blue **(N)**. Note the co-localization of BrdU and IBA1 (white arrows) and the lack of such co- localization (empty arrows). **(G,K,O)** Immunofluorescence for rat polyclonal anti-BrdU **(G,O)** in green superimposed on the rabbit polyclonal anti-GFAP **(K,O)** in red, and nuclear DAPI fluorescence in blue **(O)**. Note the co-localization of BrdU and GFAP (full arrows) and the lack of such co-localization (empty arrows). **(H,L,P)** Immunofluorescence for rat polyclonal anti-BrdU **(H,P)** in green superimposed on the guinea pig polyclonal anti-NG2 **(L,P)** in red, and nuclear DAPI fluorescence in blue **(P)**. Note the co-localization of BrdU and NG2 (full arrows) and the lack of such co-localization (empty arrows). **(I,M,Q)** Immunofluorescence for rat polyclonal anti-BrdU **(I,Q)** in green superimposed on the mouse monoclonal anti-NeuN **(M,Q)** in red, and nuclear DAPI fluorescence in blue **(Q)**. Note the high level of co-localization of BrdU and NeuN (full arrows) and the lack of such co-localization (empty arrows). Abbreviations: CTX, cerebral cortex; EC – external capsule; HIP – hippocampus; PYR, pyriform cortex; STR, striatum; THAL, thalamus. Scale bar: 126 μm.

To determine whether the neuroprotective effects of immediate curcumin treatment post HI are due to a boost of cellular proliferation, we assessed the number of BrdU positive cells in P7 naïve animals, naïve animals injected with 200 μg/g curcumin, and HI animals injected immediately post insult with either 200 μg/g curcumin or DMSO. The number of BrdU+ cells in naïve untreated animals or naïve and HI animals treated with curcumin or DMSO did not differ between the groups in any of the tested brain regions (Figure 6A). Although curcumin treatment did not result in differences in cell proliferation levels in naïve or HI animals, we assessed its effect on the proliferation of different cell types, i.e., microglia, astroglia, oligodendrocyte precursors and neurons through double labeling for BrdU and IBA1 (microglia), GFAP (astroglia), NG2 (oligodendrocyte precursors), and NeuN (neurons) and calculation of the percentage of the double positive cells over the BrdU positive ones.

Curcumin treatment of naïve or HI animals immediately after insult did not affect the percentage of IBA1 and BrdU double positive (Figure 6B), GFAP and BrdU double positive (Figure 6C), NG2 and BrdU double positive (Figure 6D), or NeuN and BrdU double positive (Figure 6E) over BrdU positive cells per region and group. The percentage of proliferating microglia (Figures 6F,N,J), astroglia (Figures 6G,O,K), and oligodendrocytes (Figures 6H,P,L) was relatively low (10–20% of the total number of BrdU positive cells) compared to the neurons which comprised 80–90% of the BrdU positive cells (Figures 6I,Q,M).

### Post-treatment With Curcumin Immediately Post-HI Decreases Phosphorylated STAT3 in Hippocampus, While Increasing Ipsilateral PHB Protein Levels at 1 h

**Figure 7:**
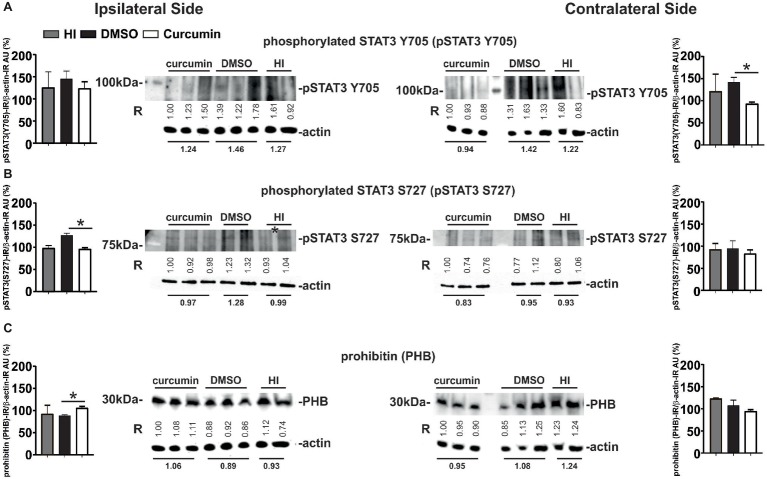
Intraperitoneal injection of curcumin (200 μg/g BW) in P7 mice immediately post-HI decreases phosphorylated STAT3 Y705 and S727 in hippocampus, while increasing ipsilateral PHB protein levels. **(A)** Western Blots for pSTAT3 (Y705) in ipsilateral and contralateral hippocampus from animals with HI, DMSO or 200 μg/g curcumin treatment and 1 h recovery. β-actin protein levels served as control. Note the increase of STAT3 Y705 ipsi- and contralaterally in the DMSO-treated group (19 and 16%, respectively) when compared to HI. Curcumin treatment decreases STAT3 Y705 protein levels ipsi- and contralaterally by 3 and 22%, respectively when compared to HI, and by 15 and 33% (*p* = 0.04) when compared to DMSO treatment. **(B)** Western Blots for pSTAT3 (S727) in ipsilateral and contralateral hippocampus from animals with HI, DMSO or 200 μg/g curcumin treatment and 1 h recovery. β-actin protein levels served as control. Note the increase of STAT3 S727 ipsi- and contralaterally in the DMSO-treated group (29 and 2%, respectively) when compared to HI. Curcumin treatment decreases STAT3 Y705 protein levels ipsi- and contralaterally by 3 and 10%, respectively when compared to HI, and by 25% (*p* = 0.009) and 12% when compared to DMSO treatment. **(C)** Western Blots for PHB in ipsilateral and contralateral hippocampus from animals with HI, DMSO or 200 μg/g curcumin treatment and 1 h recovery. β-actin protein levels served as control. Note the ipsilateral 13 and 19% (*p* = 0.0026) increase of PHB protein levels in the curcumin treated animals compared to HI and DMSO, respectively. Interestingly curcumin treatment results in contralateral 23 and 12% decrease of PHB protein levels compared to HI and DMSO littermates, respectively. PHB levels in DMSO-treated littermates were reduced by 4% in the ipsilateral and by 13% in the contralateral side compared to littermate HI animals. The ratio between pSTAT3 or PHB and β-actin immunoreactivity is shown in arbitrary units (AU) set as 100% for the first line of curcumin treated animals on each of the blots. “R” represents relative densitometry compared to β-actin, which was used as the internal loading control.

Western Blot analyses for phosphorylated STAT3 Y705 (pSTAT3 Y705) in ipsilateral and contralateral hippocampus from animals with no treatment (HI), DMSO- or 200 μg/g curcumin treatment and 1 h recovery demonstrated a slight bilateral increase in pSTAT3 Y705 in the DMSO-treated group compared to the HI controls; however, no significant differences were achieved (Figure 7A). Curcumin treatment significantly decreased pSTAT3 Y705 on the contralateral side compared to DMSO-treated animals but did not show significant reduction compared to the HI group. Curcumin treatment did not result in significant changes in the ipsilateral side (Figure 7A). Western Blot analyses for phosphorylated STAT3 S727 (pSTAT3 S727) under the same conditions showed a slight ipsilateral increase of pSTAT3 S727 in the DMSO-treated compared to HI control animals; however, significant differences were not reached (Figure 7B). Curcumin treatment significantly reduced the levels of pSTAT3 S727 compared to the DMSO-treated animals, but had no effect in comparison to the HI littermate controls. No differences between the three groups were registered on the contralateral side (Figure 7B).

Western Blots analyses for PHB at 1 h post-HI of animals with HI, DMSO, or 200 μg/g curcumin treatment showed ipsilateral increase of PHB protein levels in the curcumin compared to DMSO-treated animals, but not to HI littermate controls (Figure 7C). No significant differences were observed between the DMSO-treated animals and the HI littermate controls. No differences between the three groups were registered on the contralateral side (Figure 7C).

The values represent relative densitometry compared to β-actin, which was used as the internal loading control.

## Discussion

As shown in the current study, in a Rice-Vannucci model of severe HI insult in P7 mice, immediate, as well as delayed (60 or 120 min) application of curcumin after the HI insult clearly reduced forebrain cell death and tissue loss, as well as microglial and astroglial activation, in a dose dependent manner at 48 h post HI (Figures 2,4,5; Supplementary Figure S1). Our results show higher levels of MBP in the external capsule of the curcumin treated group at 48 h post HI and in the striatum of males at 21 days post HI, suggesting protected myelination compared to the DMSO-treated littermates and untreated HI control littermates (Figures 2N,3H). These data were in line with other studies demonstrating that decrease in MBP loss is associated with neuroprotection of white matter following neonatal HI injury (Carlsson et al., 2012; Cui et al., 2017). Our data were also in line with the results from other groups registering maintenance of myelin structure and upregulation of MBP expression in the cerebellar white matter in a model of sodium arsenite induced neurotoxicity in developing rat cerebellum (Kaushal et al., 2014). Surprisingly, we did not register similar effect in females (Figure 3I), which was also reflected in the combined (males + females) assessment (Figure 3J). This is in line with the fact that male sex is a well-established risk factor for poor neurodevelopmental outcome after birth asphyxia and that the male hippocampus, normally larger than the female, undergoes a greater volume loss compared to females (Mayoral et al., 2009). We also observed decrease in the number of iNOS positive cells in hippocampus following curcumin treatment post-HI (Figure 2O) suggesting reduction in oxidative stress. Our data is in line with previous studies in a rat model of neonatal HI where curcumin treatment prevented myelin loss, reduced iNOS expression and decreased caspase-3 dependent apoptosis (Cui et al., 2017). Additionally, our results also show reduction in TUNEL+ cell death and tissue loss (Figure 2, Supplementary Figure S1) thus broadening the spectrum of assessed cell death.

Our experiments showed attenuation of tissue loss at 21 days post HI in males, females and combined (males + females), (Figures 3A–C), however the effects were more pronounced in the male group with subregional significant differences observed in thalamus of males and combined (males + females) (Figures 3E,G). The levels of damage in the female group were lower compared to the males (Figure 3D). Sex-related susceptibility to brain damage is probably the reason for no visible effect of the curcumin treatment in females (Figures 3B,F). Immediate post-HI treatment with curcumin slightly improved motor balance and co-ordination, assessed through the slipping test, in each gender separately and combined; however, the differences did not prove significance. This result is in line with previous data from our group suggesting that the sensitivity of the slipping test might not be suitable for assessment of motor balance and co-ordination differences following neonatal HI (Rocha-Ferreira et al., 2018). At P28 the neonatal hippocampus is not fully developed (Semple et al., 2013) and it is possible that the damage is not reflected through motor and co-ordination challenges, thus assessments at later time points would be more indicative. Additionally, some data suggest that unilateral hippocampal damage is compensated by the contralateral healthy hippocampus, thus, obscuring behavioral outcome (Warburton et al., 2001; Jenkins et al., 2006). Therefore, more robust cognitive behavioral tests assessing short- and long-term memory post HI might prove more effective.

Curcumin is also a hormetin, thus demonstrating stimulatory properties at low doses and inhibitory ones at high doses. Therefore curcumin acts in a dose-dependent manner with lower doses being possibly more effective than higher ones, thus indicative of a hormetic response (Moghaddam et al., 2018). Dose response studies *in vivo* are essential for establishing whether a dietary factor affects organisms and cells *via* a hormetic mechanism. We tested four different doses of curcumin, i.e., 200, 50, 22, and 10 μg/g, and determined 50 μg/g as the minimal neuroprotective dose in neonatal HI brain damage (Figure 4, Supplementary Figure S1). As our data show no effect of the low doses (22 and 10 μg/g), and a protective effect at 50 and 200 μg/g, at the tested doses curcumin does not seem to exert hormetic properties when applied post injury in neonatal HI.

Curcumin pre-treatment with 100 μg/g BW significantly attenuates hypoxia-induced cerebral transvascular leakage, with concomitant downregulation in the expression of brain NFκB levels (Himadri et al., 2010). However, our results using the same pre-treatment curcumin dose but in the model of neonatal HI, increased the levels of brain damage markers (Figure 1) suggesting detrimental effect of the compound if present at the time of the insult. A possible explanation for the negative effect of curcumin in pre-treatment for HI can be attributed to the well-established anti-oxidant properties of the compound (Marchiani et al., 2014; Akter et al., 2019). Thus, when present during the HI insult in the neonate, curcumin possibly additionally reduces blood oxygen levels compared to the DMSO-treated littermates, and actually proves damaging. Interestingly, all damage markers are increased in hippocampus of the curcumin treated group, which might be due to the high metabolic rate and oxygen demand of that region (Rocha-Ferreira and Hristova, 2016). Another possibility for the contradiction in the pre-treatment results is the different developmental stage: in adults with hypoxia-induced cerebral transvascular leakage all compensatory and antioxidant pathways are well established, compared to the underdeveloped neonatal brain subjected to HI injury.

Investigating the mechanisms behind the short-term neuroprotective effects of curcumin post-treatment following neonatal HI, we explored whether the treatment had an effect on total and cell-specific proliferation. HI brain damage naturally boosts cell proliferation in the neonatal brain (Rocha-Ferreira and Hristova, 2016), however due to the magnitude of the damage, that proliferation is insufficient to provide the necessary repair. Curcumin is reported to stimulate cell differentiation (Gu et al., 2012; Mujoo et al., 2012; Chen et al., 2014). Therefore, we investigated whether the neuroprotective effects of curcumin in neonatal HI could be attributed to an increase of the levels of cell proliferation thus stimulating repair. In line with previous studies (Rocha-Ferreira and Hristova, 2016), we observed some but not significant HI-induced increase in cell proliferation compared to naïve animals, and no significant effect of curcumin treatment. Thus, we conclude that at 48 h following neonatal HI brain damage the neuroprotection provided by curcumin post-treatment is not a result of effects on cell proliferation.

The inhibitory effect of curcumin on the transcription properties of STAT3, a downstream target of IL6 as a main participant in HI brain damage (Rocha-Ferreira and Hristova, 2016), is well documented (Alexandrow et al., 2012; Devi et al., 2015; Patel et al., 2019). Previous work from our group demonstrates that phosphorylated STAT3 Y705 is bilaterally upregulated in cortex and hippocampus following neonatal HI brain damage, and its inhibition results in neuroprotection (Hristova et al., 2016). Interestingly, at 1h post-HI, we observed only contralateral significant reduction in the protein levels of phosphorylated STAT3 Y705 in hippocampus of curcumin treated animals (Figure 7A) compared to DMSO-treated littermates but not to HI controls. Similar trend is also observed in the ipsilateral hippocampus; however, the differences were not significant. Detrimental effects of DMSO treatment have previously been reported (Galvao et al., 2014) and our results suggest that DMSO treatment has a harmful effect which is counteracted by curcumin application (Figure 7A). STAT3 is an important regulator in astrocyte differentiation (Hong and Song, 2014). Although, at 1h post-HI, we observed only contralateral reduction of pSTAT3 Y705 compared to DMSO-treated animals, we registered bilateral reduction of astroglial activation following post-treatment with 200 μg/g curcumin after neonatal HI (Figures 2G,M).

Curcumin treatment in a mouse model of stroke, reduced microglial activation and the infraction volume of the injured area compared to untreated controls (Liu et al., 2017). In the same study, curcumin treatment reduced microglial gene expression of pro-inflammatory and oxidative stress markers such as TNFα, IL12 and iNOS, and *in vitro* incubation of LPS- or IFN γ activated microglia with curcumin reduced the release of pro-inflammatory cytokines (Liu et al., 2017). Our data is in line with these findings suggesting that the observed reduction in tissue loss, cell death and glial response could rely on the ability of curcumin to resolve inflammation *via* downregulation of pro-inflammatory cytokines.

A small pool of STAT3 has recently been discovered in mitochondria (mito-STAT3), regulating mitochondrial electron transport chain, affecting mitochondrial metabolism and cellular function (Yang and Rincon, 2016). Mito-STAT3 activation is mediated by Ser727 phosphorylation and has been shown to be crucial in immunological effector function and in cancer progression. Mito-Stat3 suppresses ROS formation during cancer genesis, suggesting that targeting Ser727 phosphorylation and mito-STAT3 has strong potential in treating cancer. This effect of mito-STAT3 could be critical in neonatal HI as the formation of ROS is a major factor causing cell death during the secondary energy failure (Rocha-Ferreira and Hristova, 2016), although the mechanisms of action might not be necessarily the same as in cancer genesis. Our data demonstrates ipsilateral reduction of phosphorylated STAT3 S727 in hippocampus following curcumin treatment post-HI compared to DMSO-treated animals but not to HI controls (Figure 7B) at 1h post-HI. This suggests a detrimental role of DMSO-treatment in neonatal HI brain damage, which is counteracted by curcumin. STAT3 regulates a metabolic function in mitochondria through STAT3 S727 phosphorylation, supporting Ras-dependent malignant transformation (Gough et al., 2009). Previous data from our lab have confirmed that global pharmacological inhibition of ERK phosphorylation is strongly neuroprotective in neonatal HI brain damage (Thei et al., 2018). Thus, as mito-STAT3 is a downstream target of Ras/ERK (Yang and Rincon, 2016) our data were in line with these previous studies and reduced phosphorylation of STAT3 S727 could be neuroprotective.

Our data demonstrates bilateral increase of phosphorylated STAT3 Y705 and S727 levels in the DMSO-treated animals compared to HI controls at 1h post-HI, which is in line with data from other groups reporting that low doses of DMSO induce caspase-3 independent neuronal death that involves apoptosis-inducing factor (AIF) translocation from mitochondria to the nucleus and poly-(ADP-ribose)-polymerase (PARP) activation (Galvao et al., 2014). Thus our results showing decrease of only 3% ipsi- and 22% contralaterally for STAT3 Y705, and of 3% ipsi- and 10% contralaterally for STAT3 S727 in curcumin treated compared to HI control animals is likely a result of necessity for curcumin treatment to compensate for the detrimental effects of DMSO and provide further neuroprotection.

PHB is a mitochondrial protein which has emerged as an important modulator of neuronal survival in different injury models (Zhou et al., 2012; Hernando-Rodríguez and Artal-Sanz, 2018). PHB localizes to the inner membrane of mitochondria acting as a chaperone protein, but is also found in the nucleus, where it negatively regulates transcription. PHB is significantly increased in the whole cell and markedly decreased in the nuclear matrix after curcumin treatment of HaCaT cells (Yang et al., 2014). Overexpression of PHB has been proven neuroprotective in a mouse model of middle cerebral artery occlusion (Kahl et al., 2018). Knocking down PHB by siRNA partly increased the apoptosis level of the neuronal cell line PC12 stimulated by H_2_O_2_ (Xu et al., 2014). Our results at 1h post-HI show ipsilateral increase of the protein levels of PHB in hippocampus following curcumin treatment post-HI (Figure 7C). This is in line with the effects observed by other groups, generally associating increased PHB expression with decrease of cell death, amelioration of mitochondrial dysfunction and neuroprotection. However, it is unclear whether, similarly to STAT3 Y705 (Hristova et al., 2016) PHB plays a different role in different cell types involved in neonatal HI, and whether its expression is time-dependent.

In conclusion, our data support a dose-dependent neuroprotection provided by immediate and delayed treatment with curcumin following neonatal HI injury. The precise mechanism of this protection is unclear; however, our results show effects of curcumin on oxidative stress and myelination, inflammation and transcription (STAT3 Y705) and mitochondrial dysfunction (STAT3 S727 and PHB). This makes curcumin an attractive therapeutic candidate in neonatal HI induced brain damage.

Although phase I clinical trials have shown that curcumin is safe even at high doses (12 g/day), its future use and clinical application is limited as a result of its poor bioavailability due to poor absorption, rapid metabolism, and rapid systemic elimination (Anand et al., 2007; Gupta et al., 2013). Therefore, future experiments should focus on increasing curcumin bioavailability and solubility with the development of aqueous solutions for clinical application.

## Ethics Statement

All animal experiments and care protocols were carried out according to the UK Animals (Scientific Procedures) Act 1986 and approved by the Home Office (PPL70/8784). The ARRIVE guidelines were followed. All experiments involved postnatal day 7 C57/Bl6 mice (P7) bred in house.

## Author Contributions

ER-F, CS, and SL contributed to the collection and processing of data, writing and editing the manuscript. SB, TF, MHa, ICR, CA, TK, and BM assisted with the collection and processing of data. DH contributed to the collection and processing of data, editing the manuscript. MHr contributed to the design of the study, collection and processing of data, writing and editing the manuscript.

### Conflict of Interest

The authors declare that the research was conducted in the absence of any commercial or financial relationships that could be construed as a potential conflict of interest.

## Supplementary Material

SUPPLEMENTARY FIGURE S1 Intraperitoneal injection of curcumin in P7 mice post-HI significantly reduces in a dose dependent manner ipsilateral brain tissue volume loss, TUNEL+ cell death and ipsilateral and contralateral reactive astrogliosis. **(A)** Intraperitoneal injection of 50 μg/g BW curcumin decreased ipsilateral volume loss compared to DMSO treated littermates, with significant individual decrease (*t*-test) in cortex (*p* = 0.018), hippocampus (*p* = 0.025) and overall (*p* = 0.0004). Mixed Linear Model treating region as a repeated measure revealed *p* = 0.0001. **(B,C)** Intraperitoneal injection of 22 μg/g **(B)** and 10 μg/g **(C)** curcumin did not have an effect on volume tissue loss compared to DMSO treated littermates. **(D)** Intraperitoneal injection of 50 μg/g curcumin decreased ipsilateral TUNEL+ cell death compared to DMSO treated littermates, with significant individual decrease (*t*-test) in hippocampus (*p* = 0.007) and external capsule (*p* = 0.0001). Mixed Linear Model treating region as a repeated measure revealed *p* = 0.016. **(E,F)** Intraperitoneal injection of 22 μg/g **(E)** and 10 μg/g **(F)** curcumin did not have an effect on ipsilateral TUNEL+ cell death compared to DMSO treated littermates. **(G)** Intraperitoneal injection of 50 μg/g curcumin decreased ipsilateral reactive astrogliosis assessed though OLV of GFAP immunoreactivity compared to DMSO treated littermates, with significant individual decrease (*t*-test) in pyriform cortex (*p* = 0.025), hippocampus (*p* = 0.033), thalamus (*p* = 0.030) and external capsule (*p* = 0.031). Mixed Linear Model treating region as a repeated measure revealed *p* = 0.014. **(H,I)** Intraperitoneal injection of 22 μg/g **(H)** and 10 μg/g **(I)** curcumin did not have an effect on ipsilateral TUNEL+ cell death compared to DMSO treated littermates. **(J)** Intraperitoneal injection of 50 μg/g curcumin reduced contralateral reactive astrogliosis compared to DMSO treated littermates, with significant individual decrease (*t*-test) in pyriform cortex (*p* = 0.009). Mixed Linear Model treating region as a repeated measure revealed *p* = 0.005. **(K,L)** Intraperitoneal injection of 22 μg/g **(H)** and 10 μg/g **(I)** curcumin did not have an effect on contralateral astrogliosis compared to DMSO treated littermates. (**A**, curcumin 50 μg/g *n* = 8, DMSO *n* = 6, **B**, curcumin 22 μg/g *n* = 12, DMSO *n* = 10, **C**, curcumin 10 μg/g *n* = 11, DMSO *n* = 11) (**p <* 0.05). Abbreviations: CTX, cerebral cortex; EC, external capsule; HIP, hippocampus; PYR, pyriform cortex; STR, striatum; THAL, thalamus.
