# Supplementary figures and images for: Curcumin: Novel Treatment in Neonatal Hypoxic-Ischemic Brain Injury
Source: Front Physiol. 2019 Nov 13;10:1351. doi: 10.3389/fphys.2019.01351 (PMC6863777; doi:10.3389/fphys.2019.01351)

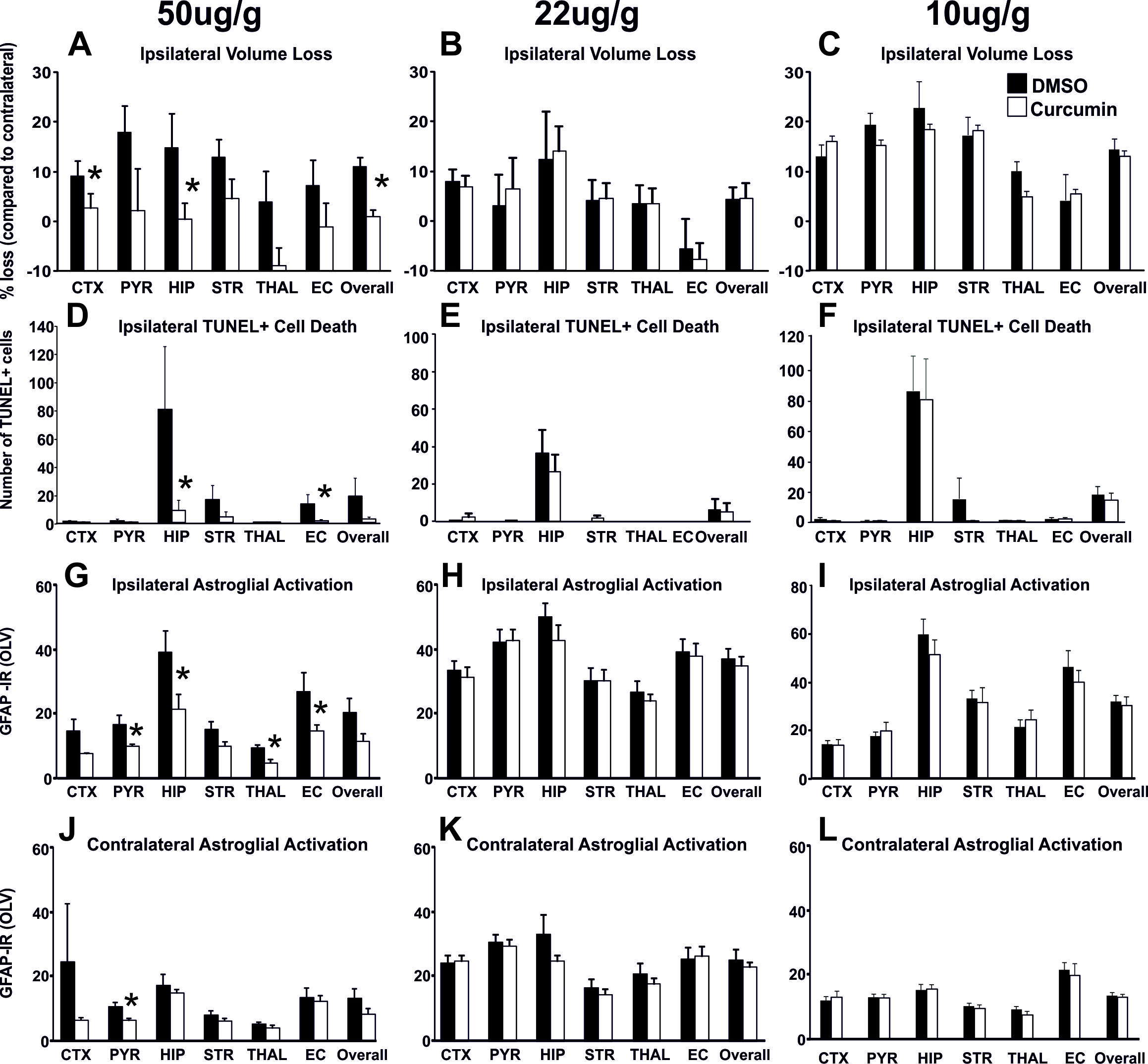

Supplement: SUPPLEMENTARY FIGURE S1 — Intraperitoneal injection of curcumin in P7 mice post-HI significantly reduces in a dose dependent manner ipsilateral brain tissue volume loss, TUNEL+ cell death and ipsilateral and contralateral reactive astrogliosis. (A) Intraperitoneal injection of 50 μg/g BW curcumin decreased ipsilateral volume loss compared to DMSO treated littermates, with significant individual decrease (t-test) in cortex (p = 0.018), hippocampus (p = 0.025) and overall (p = 0.0004). Mixed Linear Model treating region as a repeated measure revealed p = 0.0001. (B,C) Intraperitoneal injection of 22 μg/g (B) and 10 μg/g (C) curcumin did not have an effect on volume tissue loss compared to DMSO treated littermates. (D) Intraperitoneal injection of 50 μg/g curcumin decreased ipsilateral TUNEL+ cell death compared to DMSO treated littermates, with significant individual decrease (t-test) in hippocampus (p = 0.007) and external capsule (p = 0.0001). Mixed Linear Model treating region as a repeated measure revealed p = 0.016. (E,F) Intraperitoneal injection of 22 μg/g (E) and 10 μg/g (F) curcumin did not have an effect on ipsilateral TUNEL+ cell death compared to DMSO treated littermates. (G) Intraperitoneal injection of 50 μg/g curcumin decreased ipsilateral reactive astrogliosis assessed though OLV of GFAP immunoreactivity compared to DMSO treated littermates, with significant individual decrease (t-test) in pyriform cortex (p = 0.025), hippocampus (p = 0.033), thalamus (p = 0.030) and external capsule (p = 0.031). Mixed Linear Model treating region as a repeated measure revealed p = 0.014. (H,I) Intraperitoneal injection of 22 μg/g (H) and 10 μg/g (I) curcumin did not have an effect on ipsilateral TUNEL+ cell death compared to DMSO treated littermates. (J) Intraperitoneal injection of 50 μg/g curcumin reduced contralateral reactive astrogliosis compared to DMSO treated littermates, with significant individual decrease (t-test) in pyriform cortex (p = 0.009). Mixed Linear Model treating region as [file Image_1.jpg]
